# Breathing the air of mitochondrial respiration via an important oncotarget - mitochondrial glycerophosphate dehydrogenase (mGPDH)

**DOI:** 10.18632/oncotarget.27292

**Published:** 2019-11-05

**Authors:** Shilpa Thakur, Joanna Klubo-Gwiezdzinska

**Keywords:** mGPDH, thyroid cancer, metformin, metabolism

Targeting cells metabolism has been a promising therapeutic approach for cancer management. Mitochondria play a central role in the metabolism of cancer cells. Cancer cells modulate their metabolism to support their rapid proliferation. According to the Warburg effect, cancer cells shift their metabolism from oxidative phosphorylation (OXPHOS) to glycolysis even under normoxic conditions. However, cancer metabolism cannot be solely explained by this simplistic model. Studies have shown metabolic heterogeneity within the tumor cells, with some undergoing glycolysis, while others relying on OXPHOS for energy production. According to the Reverse-Warburg effect, which was introduced in the last decade, stromal cells as well as subpopulations of cancer cells undergo glycolysis in order to transfer its end-products, such as lactate, to the nearby tumor cells, which utilize them to fuel OXPHOS [1]. Due to the dependence of cancer cells on OXPHOS for ATP synthesis, targeting of proteins which contribute toward OXPHOS seems promising in the field of cancer therapeutics. Mitochondrial glycerophosphate dehydrogenase (mGPDH) is a key player in the glycerophosphate shuttle that links glycolysis to OXPHOS and, as such, is a promising oncotarget in this metabolic switch [2]. mGPDH is encoded by a single gene (*GPD2*) located on chromosome 2 in humans [2]. The crystal structure of this protein is still unknown. The expression of mGPDH varies greatly among different tissues and is primarily regulated at transcriptional level.

**Figure 1:**
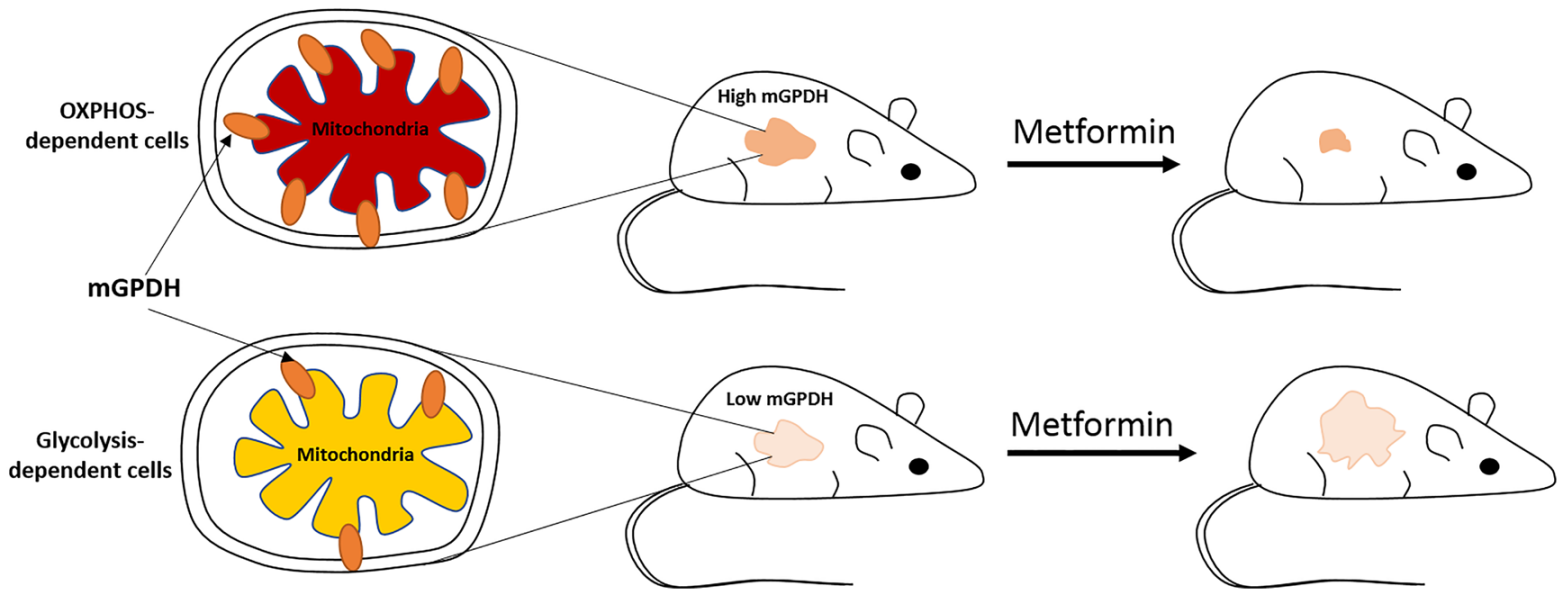
Figure 1: High mGPDH expression sensitizes thyroid cancer cells to metformin treatment. The cells characterized by high mGPDH expression have more active mitochondria in comparison to low-mGPDH expressing cells and are dependent on OXPHOS for energy generation. Metformin mediated reduction in mGPDH expression causes inhibition of OXPHOS, which in turn leads to energy stress and inhibition of cancer cell proliferation.

The *GPD2* gene is characterized by the presence of three alternate promoters which regulate mGPDH expression in a tissue-specific manner [2]. The promoter region of *GPD2* contains thyroid hormone response elements (TRE) which respond to the thyroid hormones, particularly triiodothyronine (T3), that can activate mGPDH transcription in a tissue-specific manner. This information is particularly important since T3 has been shown as cancer growth promoting factor in many cancer models, including breast, prostate, thyroid, lungs and renal cancer [3]. In fact, in a recent study, we observed T3-mediated upregulation in mGPDH expression in human thyroid cancer cells [4]. The role of mGPDH in cancer cell metabolism and tumor progression has not been exploited much. Besides thyroid cancer, higher mGPDH expression has been observed in prostate cancer cell lines as well as in the liver cancer [5]. In the latter, it has been associated with a poor prognosis, per the Protein Atlas. Among the different compounds tested, a benzoxadiazole-derivative (compound 5108184) has been found to be the most effective in inhibiting mGPDH and the prostate cancer cell line growth. In addition, benzimidazole derivatives (compounds RH02211, iGP-1, iGP-5) have also been shown as potent inhibitors of mGPDH [6, 7]. Interestingly, in the recently published study we reported mGPDH as a target of an antidiabetic drug - metformin [4]. Metformin is a safe and well-tolerated drug associated with improved response to cancer therapy in many human cancer models. In our study, we demonstrated that the level of mGPDH expression determines the efficacy of metformin treatment in metastatic tumors derived from thyroid cancer cells (Figure 1). This observation was important as we have shown that thyroid cancer tissues are characterized by higher mGPDH expression in comparison to the normal thyroid. Utilization of two different thyroid cancer cell lines characterized by different level of mGPDH expression revealed that cells with higher mGPDH expression were OXPHOS dependent, whereas cells with low-level mGPDH expression were primarily glycolysis-dependent. These observations were consistent with the role of mGPDH in OXPHOS. Moreover, metastatic tumors developed using the thyroid cells with high mGPDH expression responded well to the metformin treatment in comparison to the tumors developed from low-mGPDH expressing cells. These observations were further supported by functional studies involving mGPDH silencing and overexpression in thyroid cancer cell line models [4]. This suggests that the thyroid cancer patients characterized by higher mGPDH expression might be good candidates for metformin therapy. In fact, the anti-cancer effects of metformin have been observed in diabetic patients with thyroid cancer [8]. Studies have shown a reduction in the size of thyroid nodules, better remission rate and reduced thyroid cancer risk in diabetic patients treated with metformin in comparison with non-metformin treated diabetic patients. These patient-based observations have been further supported by various *in vitro* and *in vivo* studies, where metformin treatment inhibited thyroid cancer cells metabolism and proliferation associated with inhibition of tumor progression [8].

mGPDH has also been reported as a metformin target in the liver where suppression of mGPDH enzymatic activity by metformin leads to an alteration in cellular redox state and inhibition of hepatic gluconeogenesis in rats [9].

In addition to its role in cancer and liver metabolism, mGPDH has been observed to play a role in regulation of inflammatory responses. A recent study showed that mGPDH-mediated regulation of glucose oxidation contributes towards the regulation of inflammatory responses by controlling the production of acetyl coenzyme A (acetyl-CoA). Acetyl-CoA is a substrate for histones acetylation and regulation of its level by mGPDH has been shown to directly impact the expression of inflammatory genes which are regulated by histones acetylation [10]. mGPDH has also been shown to play a role in regulation of cellular senescence. mGPDH is a downstream mediator of IMMP2L (Inner Mitochondrial Membrane Peptidase Subunit 2) which is a central regulator of senescence process. Knockdown of mGPDH has been shown to induce senescence in fetal lung fibroblasts [11]. Cellular senescence is a double-edged sword which can promote cancer development as well as can protect a cell from malignant transformation. It will be interesting to evaluate the role of mGPDH in regulation of senescence within cancer cells.

Despite its importance in metabolism, the expression and role of mGPDH has not been explored much in different cancers. In our study, we provided the evidence that mGPDH is a metformin target in thyroid cancer cells and its expression determines the efficacy of metformin treatment in metastatic tumors derived from thyroid cancer cells. In the future, it will be interesting to evaluate the growth inhibitory effects of metformin as well as other mGPDH inhibitors in tumors expressing high levels of mGPDH and to test its role in cancer metabolism, tumor inflammation and senescence.
